# Prenatal Valproate Exposure Differentially Affects Parvalbumin-Expressing Neurons and Related Circuits in the Cortex and Striatum of Mice

**DOI:** 10.3389/fnmol.2016.00150

**Published:** 2016-12-21

**Authors:** Emanuel Lauber, Federica Filice, Beat Schwaller

**Affiliations:** Anatomy, Department of Medicine, University of FribourgFribourg, Switzerland

**Keywords:** ASD, VPA, parvalbumin, K_v_3, HCN, excitation/inhibition balance

## Abstract

Autism spectrum disorders (ASD) comprise a number of heterogeneous neurodevelopmental diseases characterized by core behavioral symptoms in the domains of social interaction, language/communication and repetitive or stereotyped patterns of behavior. *In utero* exposure to valproic acid (VPA) has evolved as a highly recognized rodent ASD model due to the robust behavioral phenotype observed in the offspring and the proven construct-, face- and predictive validity of the model. The number of parvalbumin-immunoreactive (PV^+^) GABAergic interneurons has been consistently reported to be decreased in human ASD subjects and in ASD animal models. The presumed loss of this neuron subpopulation hereafter termed Pvalb neurons and/or PV deficits were proposed to result in an excitation/inhibition imbalance often observed in ASD. Importantly, loss of Pvalb neurons and decreased/absent PV protein levels have two fundamentally different consequences. Thus, Pvalb neurons were investigated in *in utero* VPA-exposed male (“VPA”) mice in the striatum, medial prefrontal cortex (mPFC) and somatosensory cortex (SSC), three ASD-associated brain regions. Unbiased stereology of PV^+^ neurons and Vicia Villosa Agglutinin-positive (VVA^+^) perineuronal nets, which specifically enwrap Pvalb neurons, was carried out. Analyses of PV protein expression and mRNA levels for *Pvalb, Gad67, Kcnc1, Kcnc2, Kcns3, Hcn1, Hcn2, and Hcn4* were performed. We found a ∼15% reduction in the number of PV^+^ cells and decreased *Pvalb* mRNA and PV protein levels in the striatum of VPA mice compared to controls, while the number of VVA^+^ cells was unchanged, indicating that Pvalb neurons were affected at the level of the transcriptome. In selected cortical regions (mPFC, SSC) of VPA mice, no quantitative loss/decrease of PV^+^ cells was observed. However, expression of *Kcnc1*, coding for the voltage-gated potassium channel K_v_3.1 specifically expressed in Pvalb neurons, was decreased by ∼40% in forebrain lysates of VPA mice. Moreover, hyperpolarization-activated cyclic nucleotide-gated channel (HCN) 1 expression was increased by ∼40% in the same samples from VPA mice. We conclude that VPA leads to alterations that are brain region- and gene-specific including *Pvalb, Kcnc1*, and *Hcn1* possibly linked to homeostatic mechanisms. Striatal PV down-regulation appears as a common feature in a subset of genetic (Shank3B-/-) and environmental ASD models.

## Introduction

Autism Spectrum Disorders (ASD) are a group of neurodevelopmental disorders that share core behavioral symptoms in the domains of social interaction, language/communication and repetitive, or stereotyped patterns of behavior (American Psychiatric Association, 2013). The etiology of ASD is still poorly understood; ASD is viewed as a multifactorial disease, caused by a combination of genetic, epigenetic, and environmental cues. The genetics of ASD are extremely heterogeneous with a large number (>100) of identified risk genes, yet mutations in one of these risk genes occur sporadic and do not affect more than 1–2% of ASD cases (Kleijer et al., 2014; de la Torre-Ubieta et al., 2016). Environmental insults during embryonic development and early postnatal life are thus considered to play an important role in ASD pathophysiology.

Valproic acid (VPA; also known as valproate) is used in clinics for the treatment of epilepsy and psychiatric conditions such as bipolar disorders and acute mania (Haddad et al., 2009). Epidemiological studies in children have shown a positive correlation between *in utero* VPA exposure and the diagnosis of ASD (Bromley et al., 2013; Christensen et al., 2013). VPA monotherapy during pregnancy results in about seven-fold greater incidence of ASD or ASD key symptoms including language impairment, reduced attention, social deficits and restricted interests (Vinten et al., 2009). The effects of VPA are thought to be induced by a broad range of molecular mechanisms including: inhibition of histone deacetylation (HDAC) (Phiel et al., 2001; Gottfried et al., 2013), inositol depletion (Eickholt et al., 2005), increase in fetal oxidative stress (Verrotti et al., 2008), changes in gene expression (Ornoy, 2009) and induction of GABA synthesis (Loscher, 1999). VPA exposure during pregnancy has been extensively studied in rodents and has evolved as the well-established “VPA mouse or rat model” for the study of ASD. Behavioral phenotypes related to all human core symptoms of ASD including impaired social behavior, repetitive or stereotyped patterns of behavior and impaired communication exist in juvenile VPA rats and mice and persist into adulthood (reviewed in Roullet et al., 2013; Ergaz et al., 2016). The striking and robust ASD phenotype, together with the given construct validity, has made it attractive for further studying the pathophysiology of ASD.

Amongst other morpho-functional abnormalities, VPA mice or rats were reported to exhibit a loss of PV-immunoreactive (PV^+^) neurons in “PV-empty zones”, i.e., patchy zones devoid of PV immunoreactivity observed on sections of the neocortex (Gogolla et al., 2009); and in the colliculi superiors (Dendrinos et al., 2011). Pvalb neuronal loss and/or decreased PV expression has also been observed in post-mortem brains of human ASD patients (Zikopoulos and Barbas, 2013; Stoner et al., 2014; Hashemi et al., 2016) and various ASD mouse models (see Table 1 in Wohr et al., 2015). PV is a calcium-binding protein expressed in specific neurons in the brain (Celio, 1990) and for decades, PV has been used as a reliable marker for a subset of GABAergic inhibitory neurons in the CNS (Celio and Heizmann, 1981). In these neurons, PV serves as a slow-onset Ca^2+^ buffer modulating several Ca^2+^-dependent processes. PV^-/-^ mice show alterations in synaptic transmission including short-term plasticity, kinetics of delayed transmitter release, precision of spike timing, excitability, as well as other deviations (for review see Schwaller, 2012). Moreover, Pvalb neurons in the cortex are essential players in generating gamma band oscillations (Bartos et al., 2002; Buzsaki and Wang, 2012). Selectively reducing Pvalb neuronal activity strongly attenuates gamma oscillations, a phenomenon often observed in ASD and schizophrenia patients during cognitive tasks (Cho et al., 2006; Sohal et al., 2009). Although *Pvalb* has never been described as an ASD risk gene, PV-deficient (PV^+/-^ and PV^-/-^) mice show a striking ASD phenotype in all core domains (Wohr et al., 2015). Moreover, structural MRI revealed ASD-associated neuroanatomical changes such as transient cortical hypertrophy and cerebellar hypoplasia in PV^+/-^ and PV^-/-^ mice (Wohr et al., 2015). Importantly, Pvalb neurons in PV^+/-^ and PV^-/-^ mice (and also in the validated ASD models Shank1^-/-^ and Shank3B^-/-^ mice) are not lost, a conclusion often drawn too early when Pvalb neurons are quantified solely using PV as marker. Rather, PV expression levels might be low or absent, thus falling under detection threshold, while the Pvalb neuron number is unchanged. The latter was shown by stereological analysis of perineuronal nets (PNNs), which represent an alternative marker for Pvalb neurons or using PV-EGFP mice (Meyer et al., 2002) expressing EGFP in Pvalb neurons independent of endogenous PV expression levels (Filice et al., 2016). Here, we investigated whether Pvalb neuron numbers were altered and/or if PV expression was down-regulated in three ASD-linked brain regions, namely the mPFC, SSC and striatum of VPA male mice. Since these regions receive a vast number of sensory inputs, they are crucial for multisensory integration, executive function, language, social cognition, motivational state and regulation of emotional behavior. Alterations in the structure and function of the mPFC (Martinez-Sanchis, 2014), SSC (Markram and Markram, 2010; Khan et al., 2016) and striatum (Fuccillo, 2016) have been consistently reported in ASD. We found PV expression levels to be decreased in the striatum, but not in forebrain lysates comprising the two selected ASD-linked cortical regions. In all 3 brain regions, the number of Pvalb neurons was unchanged between VPA and control mice, strongly arguing against a loss of Pvalb neurons. VPA mice exhibited alterations in the expression of potassium channels; mRNA and protein levels of K_v_3.1, selectively expressed in Pvalb neurons, was decreased, whereas expression of hyperpolarization-activated cyclic nucleotide gated-channel (HCN) 1 was up-regulated in forebrain lysates. HCN channel-mediated I_h_ currents are impaired in Shank3^-/-^ mice (Yi et al., 2016), which exhibit striatal PV downregulation similar to VPA mice as described in this report. These changes are likely to affect the E/I balance that is often altered in the brain of ASD subjects (Uhlhaas and Singer, 2007).

## Materials and Methods

### Animals

All mice were group-housed at the University of Fribourg, Switzerland in temperature-controlled animal facilities (24°C, 12:12 h light/dark cycle), fed *ad libitum* with free access to water. C57Bl/6J mice were mated overnight until a vaginal plug was detected in the morning. The day of sperm plug detection was defined as gestational day 0 (GD0). At GD12, 600 mg/kg valproic acid sodium salt (VPA; P4543 Sigma–Aldrich, Buchs, Switzerland) diluted in 0.9% NaCl was administered by oral gavage. Control animals were administered with 0.9% NaCl. Pups were not weaned; brains were taken after cerebral dislocation at postnatal day (PND) 25 ± 1. Only male mice were used in this study. All experiments were performed with permission of the local animal care committee (Canton of Fribourg, Switzerland) and according to the present Swiss law and the European Communities Council Directive of 24 November 1986 (86/609/EEC).

### Tissue Preparation and Immunohistochemistry

Mice at PND25 were anesthetized (Esconarkon, Streuli Pharma AG, Uznach, Switzerland) and perfused with 0.9% saline solution followed by 4% PFA. Brains were removed and post-fixed for 24 h in 4% PFA before being processed in 30% sucrose-TBS at 4°C. After cryopreservation, coronal sections were cut rostro-caudally using a freezing microtome (Frigomobil, Reichert-Jung, Vienna, Austria); six series of equidistant sections were collected using stereological systematic random sampling principles (see below). Free-floating sections were initially blocked with TBS 0.1 M, pH 7.3 plus 0.4% Triton X-100 and 10% bovine serum albumin (BSA) for 1 h at room temperature. Next, sections were washed three times with TBS, and incubated with PV antibody (anti-rabbit PV25, Swant, Marly, Switzerland) at a dilution of 1:1000 and with Vicia Villosa Agglutinin (biotinylated-VVA, Reactolab, Servion, Switzerland) at a concentration of 10 μg/ml in TBS containing MgCl_2_, MnCl_2_, CaCl_2_ (final salt concentration: 0.1 mM each) overnight at 4°C. Sections were rinsed once with TBS, then twice with Tris-HCl 0.1 M, pH 8.2; they were then incubated (protected from light) at room temperature with anti-rabbit Cy3-conjugated antibody (1:200 dilution) and Cy2 streptavidin-conjugated antibody (at a dilution of 1:200, from Milan Analytic AG, Switzerland) in Tris-HCl. DAPI staining allowed to visualize nuclei of fixed cells (1:1000 dilution, LuBio Science GmbH, Luzern, Switzerland) in PBS 0.1 M, pH 7.4. After final rinsing, slides were coverslipped with Hydromount (National Diagnostics, Atlanta, GO, USA).

### Stereological Quantification

We used the optical fractionator method (West et al., 1991) to estimate the total number of PV-positive (PV^+^) and Vicia Villosa Agglutinin-binding (VVA^+^) neurons in brain regions of interest (ROIs) using the Stereo Investigator system (Version 11, MicroBrightField, Williston, VT, USA). The Stereo Investigator system was connected to a Zeiss Axioplan microscope with a motorized x-y stage (Ludl Electronic Products, LTD, NY, USA) and coupled to a Hamamtsu Orca Camera. ROIs were defined based on stereotactic coordinates provided by the Paxinos and Franklin atlas (Paxinos, 2001). The mPFC was defined at 1.94 to 1.10 mm from bregma comprising the anterior cingulate cortex, prelimbic area and infralimbic area; the corpus callosum, the midline between the hemispheres and fissura longitudinalis cerebri served as borders. The striatum (caudoputamen) was defined at 1.10 to –0.82 mm from bregma; the lateral ventricle, corpus callosum, capsula externa and globus pallidus externum served as borders, whereas the commisura anterior and rhinal fissure served as reference points. The SSC was defined at 1.94 to –1.82 mm from bregma; the corpus callosum and capsula externa served as borders, whereas the lateral ventricle, cingulum, hippocampus and outer brain curvature served as reference points. Cell counting was carried out on images obtained with oil immersion objective lenses (100x; NA = 1.40 and 63x; NA = 1.30). The volume of the analyzed brain structure was determined using the Cavalieri estimator (Gundersen et al., 1988). Five animals were analyzed per group, with pups from at least 3 different litters for each group. All results obtained from stereological quantification are reported in **Table 3**.

### Counting Criteria

**FIGURE 1:**
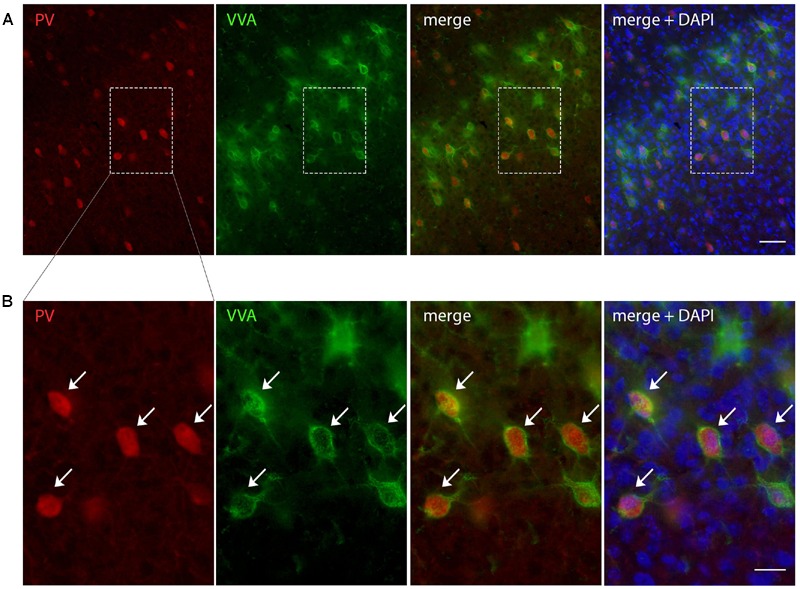
**Representative images of PV and VVA co-localization from PND25 mouse cortex. (A)** PV^+^ cells (red) are enwrapped by VVA^+^ perineuronal nets (green). Slices were counterstained with DAPI (blue). **(B)** Higher magnification of the boxes outlined in A. Arrowheads point at the PV^+^VVA^+^ double-positive cells that are in focus. Scale bar: **(A)** 50 μm; **(B)** 20 μm.

Sampling parameters are reported in **Table 1**. VVA^+^ and PV^+^ cells were counted independently and according to the following criteria: (1) Well visible DAPI-stained nucleus; (2) well-defined perineuronal net (PNN) with a web-/lattice-like morphology for VVA^+^ cells; examples are shown in **Figure 1**. (3) PV staining surrounding the DAPI-stained nucleus for PV^+^ neurons. The thickness of individual sections was measured at every fifth sampling location, and the mean of all measurements was used for all computations. At each selected location, the microscope was focused down through the disector sample in order to count any positive cell within that particular counting frame according to disector counting rules. Of importance, the cell number estimate is legitimate, even if the tissue volume changes during processing, because the fractionator method does not necessitate a measurement of tissue volume or any other dimensional quality. The total number of cells (N) in the selected ROIs was estimated as summarized by West et al. (1991, 1996) using the equation:

**Table 1:** Stereological sampling parameters.

Brain region	Cutting plane	No. of sections	Section evaluation interval (μm)	Height of disector (μm)	Guard zone (μm)	Counting frame area (μm)	Sampling grid area (μm)	Measured section thickness mean (μm)	Cavalieri grid size (μm)
Striatum	Coronal	6–7	6	20.0	0.5	110 × 90	350 × 350	21.06	150 × 150
SSC	Coronal	14–15	6	20.0	0.5	70 × 55	450 × 450	21.76	200 × 200
mPFC	Coronal	4	6	20.0	0.5	90 × 70	150 × 200	20.80	100 × 100

$$\text{N}\;=\;\sum Q^{-}\;\times \;\left(\frac{1}{ssf}\right)\;\times \;\left(\frac{1}{asf}\right)\;\times \;\left(\frac{1}{tsf}\right)$$

where Q^-^ signifies the number of objects (counts), and *ssf, asf*, and *tsf* correspond to the section sampling fraction, the area sampling fraction and the thickness sampling fraction, respectively. The accuracy of the estimates N is defined by the coefficient of error CE (**Table 1**), which represents the sampling error related to the counting noise, systematic uniform random sampling and variances in section thickness (Gundersen et al., 1999). For most biological samples, a CE value of 0.10 is largely accepted. CEs for both *m* = 1 and *m* = 0 are provided in **Table 1** and are expected to bracket the true CE values of the estimates. In our double-labeling experiments, strictly individual fluorescence images for either PV^+^ or VVA^+^ structures were counted as reported before (Filice et al., 2016) without crosschecking the other channel. This ascertained that a cell (or a small part of a cell resulting from the sectioning) with “weak” staining intensity (i.e., below the predefined threshold) and/or characterized by an “atypical” shape (not easily discernable as cell-like) was considered negative, even if a check of the other channel would have probably identified this cell/cell segment as positive for the second marker.

### RT-qPCR

Mice were euthanized by cervical dislocation and the brain was quickly removed and put in ice-cold 0.9% saline solution. The brain was cut in half along the midline and the cerebellum was removed. The hippocampus and striatum were dissected by carefully removing (pulling) these structures as described in Supplementary Figure S2. The remaining parts of the brain consisting essentially of cortex (approximately 80% of the total volume) and to minor extent (<20%) subcortical structures including thalamus and pallidum were collected as “forebrain” samples. All tissue samples were snap-frozen in liquid nitrogen and stored at –80°C for further use. The left hemisphere was always dissected first and used for qRT-PCR. Total RNA was extracted from mouse brain tissue (striatum and “forebrain” from the left hemisphere) using the peqGold TRIzol reagent (Peqlab, VWR International GmbH, Erlangen, Germany). cDNA was synthesized using ThermoFisher’s Verso cDNA Synthesis Kit (ThermoFisher, Lausanne, Switzerland). qRT-PCR was carried out to examine the expression of mRNA of the *18S* rRNA, *Ubc, Pvalb, Gad67, Kcnc1, Kcnc2, Kcns3, Hcn1, Hcn2*, and *Hcn4* genes using the universal 2X KAPA SYBR FAST qPCR Master Mix (Axonlab AG, Mont-sur-Lausanne, Switzerland). Details about the primer sequences are listed in **Table 2**. Gene expression quantitation was carried out in a DNA thermal cycler (Corbett Rotor gene 6000, QIAGEN Instruments AG, Hombrechtikon, Switzerland), according to the following two-steps protocol: a denaturation step of 95°C for 3 min; 40 cycles of denaturation at 95°C for 3 s and annealing/extension/data acquisition ranging from 52 to 62°C for 20 s. The housekeeping genes 18S ribosomal RNA (*18S*) or ubiquitin C (*Ubc*) were used as endogenous controls to normalize the mRNA content for each sample. In the second cohort of animals, *Ubc* mRNA signals were found to show lower variability (smaller S.D.) compared to *18S*, both within individual animals and within the groups (VPA vs. control, data not shown). Thus *Ubc* was used to normalize levels of *Kcnc2, Kcns3, Gad67, Hcn1, Hcn2*, and *Hcn4* mRNA levels in the striatum of these animals. However, normalization with *18S* mRNA levels resulted in essentially similar values. mRNA levels were quantified by the 2^-ΔΔCt^ method and normalized to (I) housekeeping genes and furthermore (II) to the control mice group as described before (Livak and Schmittgen, 2001).

**Table 2:** qRT-PCR primers.

Primer	Sequence 5′–3′	Nt position	Gene	Gene accession number
Pvalb	For: TGTCGATGACAGACGTGCTC Rev: TTCTTCAACCCCAATCTTGC	24–43 309-328	*Pvalb*	NM_013645
18S rRNA	For: TCAAGAACGAAAGTCGGAGGTT Rev: GGACATCTAAGGGCATCACAG	1026–1047 1493-1513	*Rn18S*	NR_003278
UBC	For: CGGAGTCGCCCGAGGTCACA Rev: CTGCATCGTCTCTCTCACGGAGTT	23–42 94-117	*Ubc*	NM_019639
GAD67	For: AATCTTGCTTCAGTAGCCTTCG Rev: TGTCTTCAAAAACACTTGTGGG	2979–3000 3178–3199	*Gad1*	NM_001312900
K_v_3.1	For: GTGCCGACGAGTTCTTCTTC Rev: GTCATCTCCAGCTCGTCCTC	1362–1381 1646–1665	*Kcnc1*	NM_001112739
K_v_3.2	For: AGATCGAGAGCAACGAGAGG Rev: GGTGGCGATCGAAGAAGAAT	72–91 379–398	*Kcnc2*	NM_001025581
K_v_9.3	For: CCCTGGACAAGATGAGGAAC Rev: TTGATGCCCCAGTACTCGAT	465–484 745–764	*Kcns3*	NM_173417
HCN1	For: CTCAGTCTCTTGCGGTTATTACG Rev: TGGCGAGGTCATAGGTCAT	1138–1160 1210–1228	*Hcn1*	NM_010408
HCN2	For: ATCGCATAGGCAAGAAGAACTC Rev: CAATCTCCTGGATGATGGCATT	1936–1957 2017–2037	*Hcn2*	NM_008226
HCN4	For: GCATGATGCTTCTGCTGTGT Rev: GCTTCCCCCAGGAGTTATTC	1268–1287 1371–1390	*Hcn4*	NM_001081192

### Western Blot Analyses

Frozen brain tissue from the dissected second (right) hemispheres as described above were homogenized and soluble or membrane proteins extracted for Western blotting experiments as described before (Racay et al., 2006; Maetzler et al., 2009). Denatured proteins (30 μg) were separated by SDS-PAGE (10–12.5%). After electrophoresis, the proteins were transferred on nitrocellulose membranes (MS solution, Chemie Brunschwig, Basel, Switzerland). The membranes were then blocked with 5% bovine serum albumin (BSA) in TBS for 60 min at room temperature and incubated with primary antibodies: rabbit anti-PV25 (Swant, Marly, Switzerland), rabbit anti-GAPDH (Sigma–Aldrich, Buchs, Switzerland) diluted 1:10,000, rabbit anti-K_v_3.1b (Merck Millipore, Schaffhausen, Switzerland), rabbit anti-HCN1 (Alomone Labs, Jerusalem, Israel) diluted 1:200 in 2% BSA in TBS-T overnight at 4°C. Membranes were washed three times in TBS-T and incubated for 1 h with secondary antibody (goat anti-rabbit IgG HRP-conjugated, Sigma–Aldrich, Buchs, Switzerland) diluted at 1:10,000 in TBS-T. Finally, membranes were repeatedly rinsed in TBS-T and developed using ECL (Merck Millipore, Schaffhausen, Switzerland). Bands visualized by ECL were quantified using Image Studio Light Version 5.0. GAPDH signals were used as loading control.

### Statistical Analysis and Cell Number Estimates

Stereological data, mRNA and protein levels were compared between groups by the Student’s *t*-test. Data were analyzed using the GraphPad Prism 6 software (San Diego, USA). As no significant differences were observed when comparing ROIs in the two hemispheres of the same mouse, analyses were carried out with the pooled data of both hemispheres. The morphological data were initially checked for normal distribution by the Kolmogorov–Smirnov test and further analyzed with the Student’s *t*-test. A *p*-value < 0.05 was considered statistically significant.

## Results

### VPA-Treated Mice are Healthy and Develop Normally

Different protocols for the VPA animal model of ASD have been previously applied. We chose to use a dose of 600 mg/kg administered at GD12, because of the well-described behavioral phenotype observed in these mice (Gottfried et al., 2013; Mabunga et al., 2015). Oral gavage was performed to accurately control the dose and minimize risk to harm the pregnant females or the fetus. We did not detect any physical malformation or conspicuous features in the pups from VPA-treated mothers, except for five pups from two different litters that manifested few hairless spots at PND15. However, occurrence of hair loss is a characteristic of the C57Bl/6J and related mouse strains. Litter size, sex distribution and weight of the pups were not different between VPA and control mice (Supplementary Figure S1).

### PV Expression Levels, But Not Pvalb Neuron Numbers are Decreased in the Striatum of VPA Mice

To evaluate the involvement of Pvalb neurons in the VPA mouse model, we performed stereology-based analysis of VPA mice at PND25 ± 1. The optical fractionator method was used to reliably quantify cell numbers in three ASD-associated brain regions, namely the striatum, medial prefrontal cortex (mPFC) and somatosensory cortex (SSC). Coefficient of error (CE) values ranged from 0.06 to 0.11 (**Table 3**) indicating a high precision of the estimates for both PV^+^ and VVA^+^ populations (Gundersen et al., 1999). One of the most crucial points when counting PV^+^ cells stained with an anti-PV antibody, is, whether the absence of a signal signifies “Pvalb neuron loss” or rather “PV down-regulation”. Therefore, in addition to counting PV^+^ cells via anti-PV immunostaining, we simultaneously quantified the number of Vicia Villosa Agglutinin VVA^+^ cells, i.e., neurons surrounded by PNNs in the same brain regions to obtain an alternative estimate for the number of Pvalb cells. VVA is a lectin that binds to *N*-acetylgalactosamine residues of PNNs, which specifically surround Pvalb neurons (Hartig et al., 1992; Haunso et al., 2000), as also shown in our previous study (Filice et al., 2016). VVA^+^ cells were considered to serve as a correlate for Pvalb neurons; a typical example of PV and VVA co-localization is shown in **Figure 1**. In the selected cortical region, essentially all PV^+^ cells are surrounded by a PNN identified as VVA^+^ cells. The one cell showing strong PNN labeling, but weak to none PV staining was mostly out of focus and cut very tangentially, i.e. containing a minimal part of the somatic region within the section, also evidenced by the low to absent DAPI staining.

**Table 3:** Mean total number of PV^+^ and VVA^+^ cells in the striatum, SSC and mPFC of saline- and VPA-exposed mice.

	Striatum PV^+^				Striatum VVA^+^			
	Mean	*SD*	*P*-value	CE_m = 0_/_m = 1_ ≤	Mean	*SD*	*P*-value	CE_m = 0_/_m = 1_ ≤
Control VPA	21′251 18’090	1051 1790	0.0093	0.09/0.06 0.10/0.07	26’518 27’225	460 3076	0.6249	0.09/0.06 0.09/0.06
	
	**SSC PV^+^**				**SSC VVA^+^**			
	**Mean**	**SD**	***P*-value**	**CE_m = 0_/_m = 1_ ≤**	**Mean**	**SD**	***P*-value**	**CE_m = 0_/_m = 1_ ≤**
	
Control VPA	121′971 128′896	4987 14′897	0.3532	0.07/0.06 0.08/0.06	143’397 153’087	8093 13’281	0.2010	0.06/0.05 0.08/0.07
	
	**mPFC PV^+^**				**mPFC VVA^+^**			
	**Mean**	**SD**	***P*-value**	**CE_m = 0_/_m = 1_ ≤**	**Mean**	**SD**	***P*-value**	**CE_m = 0_/_m = 1_ ≤**
	
Control VPA	6622 6760	242 1476	0.8415	0.11/0.07 0.10/0.07	7211 7380	713 1423	0.8191	0.10/0.07 0.11/0.07

**FIGURE 2:**
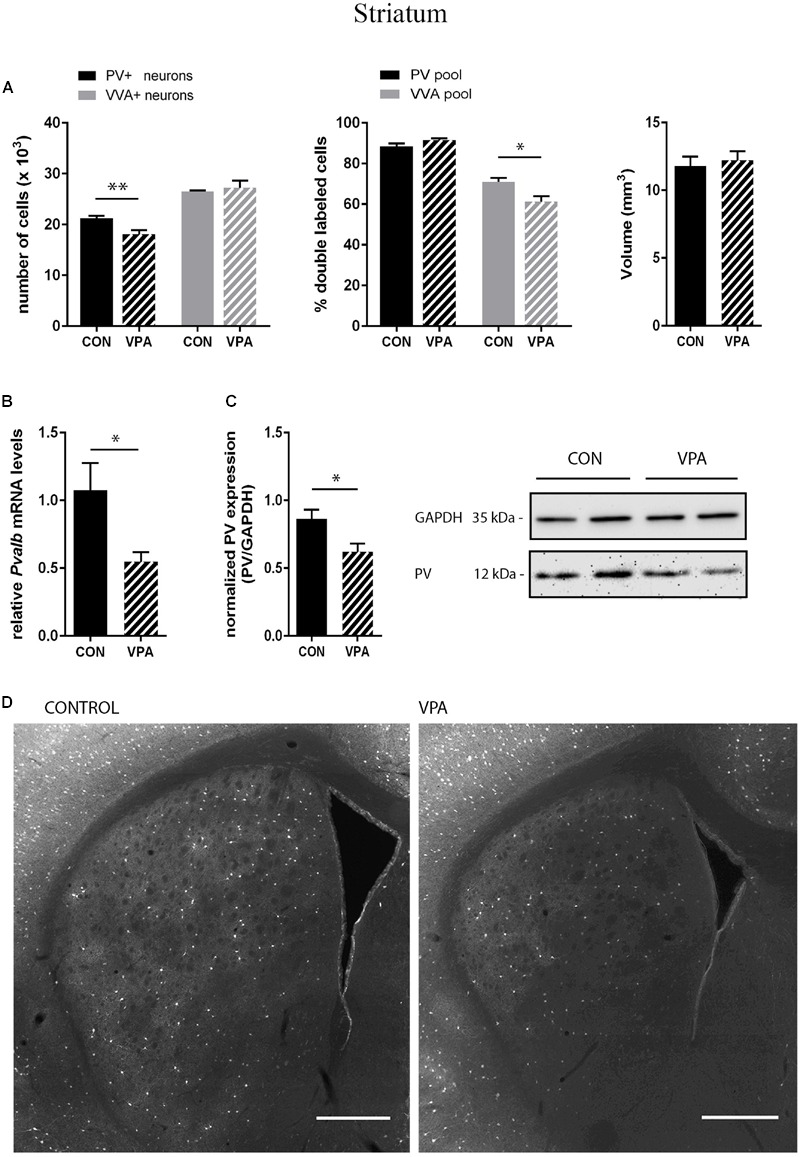
**(A)** Left: Stereological estimations of PV^+^ (black) and VVA^+^ (gray) cells in the striatum of PND25 *in utero* saline-(CON) or VPA-treated male mice (*N* = 5 each). Significant differences are observed in PV^+^ cells between CON and VPA mice (*P*-value < 0.01). Asterisks represent ^∗^*P* ≤ 0.05, ^∗∗^*P* ≤ 0.01, respectively. Middle: percentage of PV^+^ cells surrounded by VVA (black) and VVA^+^ cells showing PV expression (gray) in CON and VPA mice. Right: Volume of the ROI; i.e., the striatum. **(B)** qRT-PCR values from PND25 mice representing striatal mRNA levels were normalized to *18S* mRNA levels and expressed as fold change compared to CON (*N* = 6 mice each). **(C)** Left: Quantitative Western blot analysis of striatal samples of PND25 CON and VPA mice (*N* = 6 each). Quantification of PV protein levels in the striatum is shown. GAPDH signals served as loading controls and were used for the normalization of the PV signals. Results are expressed as a percentage of normalized PV levels measured in a mixture of lysates from six WT striatums that was loaded on all membranes. Right: Representative Western blot signals. **(D)** Representative PV immunofluorescence images from the striatum of a CON and VPA mouse are shown. Scale bar: 200 μm. All data expressed as mean ± SEM.

**FIGURE 3:**
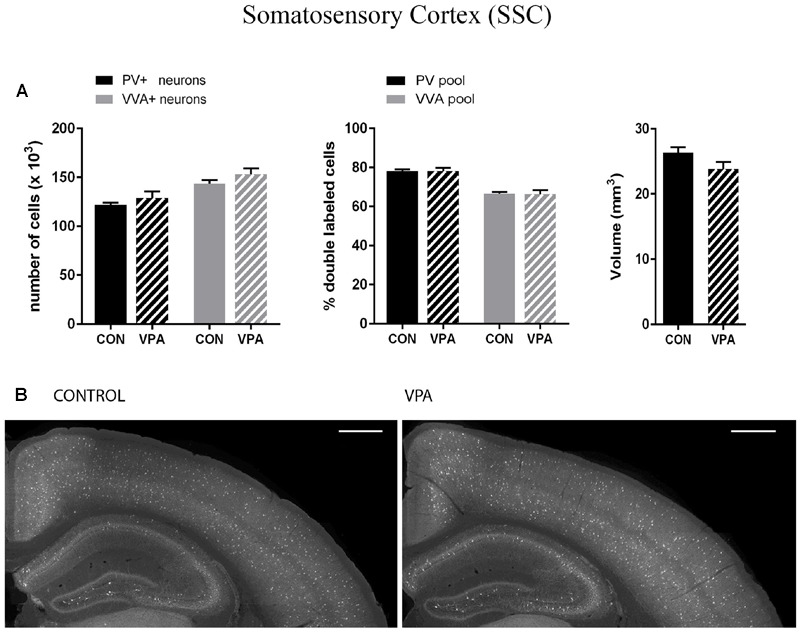
**(A)** Left: Stereological estimations of PV^+^ (black) and VVA^+^ (gray) cells in the SSC of PND25 *in utero* saline (CON) or VPA-treated male mice (*N* = 5 each). Middle: percentage of PV^+^ cells surrounded by VVA (black) and VVA^+^ cells showing PV expression (gray) in CON and VPA mice. Right: Volume of the ROI; i.e., the SSC. **(B)** Representative PV immunofluorescence images from the SSC of a CON and VPA mouse are shown. Scale bar: 200 μm. All data are expressed as mean ± SEM.

**FIGURE 4:**
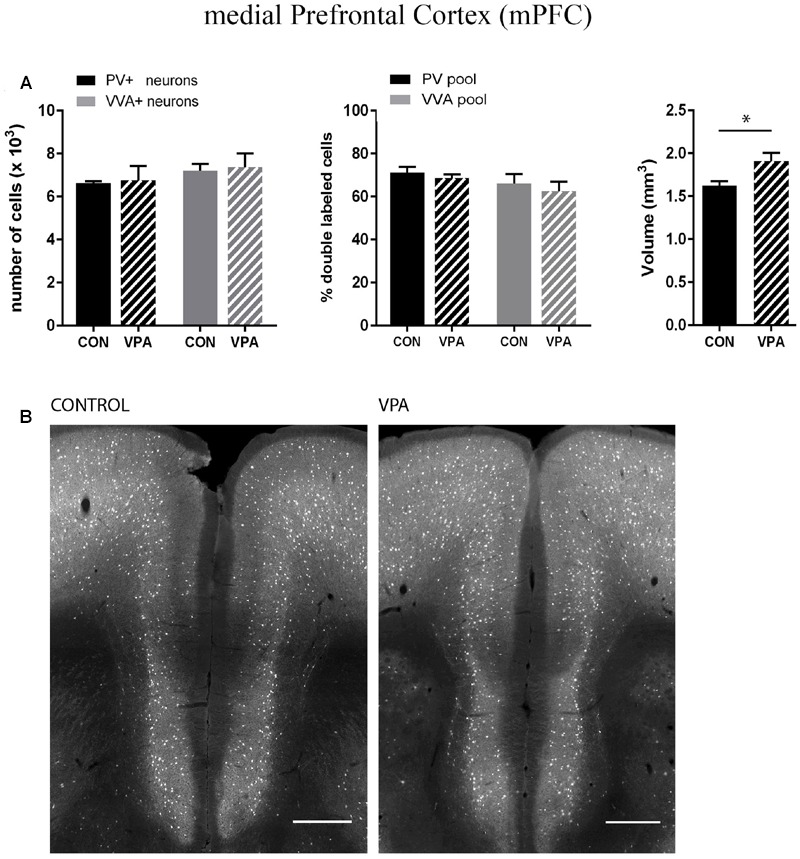
**(A)** Left: Stereological estimations of PV^+^ (black) and VVA^+^ (gray) cells in the mPFC of PND25 *in utero* saline (CON) or VPA treated male mice (*N* = 5 each). Middle: percentage of PV*^+^* cells surrounded by VVA (black) and VVA^+^ cells showing PV expression (gray) in CON and VPA mice. Right: Volume of the ROI; i.e., the mPFC. Asterisks represent ^∗^*P* ≤ 0.05. **(B)** Representative PV immunofluorescence images from the mPFC of a CON and VPA mouse are shown. Scale bar: 200 μm. All data are expressed as mean ± SEM.

**FIGURE 5:**
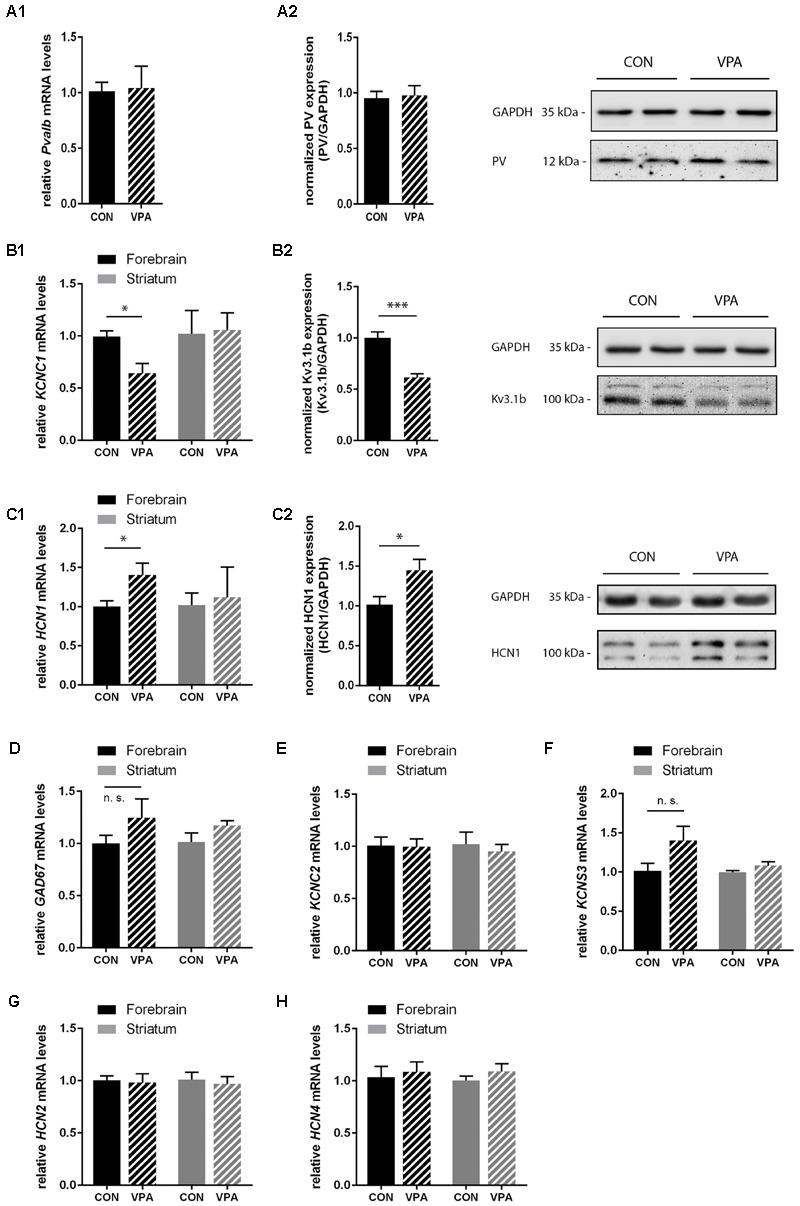
**qRT-PCR values from PND25 mice representing mRNA levels for (A1)** *Pvalb*, **(B1)** *Kcnc1*, **(C1)** *Hcn1*, **(D)** *Gad67*, **(E)** *Kcnc2*, **(F)** *Kcns3*, **(G)** *Hcn2*, and **(H)** *Hcn4* were normalized to *18S* or *Ubc* mRNA levels and expressed as fold change compared to CON (N = 5 mice each; for *Pvalb N* = 6 mice each). **(A2,B2,C2)** Quantitative Western blot analysis of forebrain samples of PND25 CON and VPA mice (*N* = 5 mice each). Quantification of PV **(A2)**, K_v_3.1b **(B2)**, and HCN1 **(C2)** protein levels in the forebrain samples are shown. Asterisks represent ^∗^*P* ≤ 0.05, ^∗∗∗^*P* ≤ 0.001. GAPDH signals served as loading controls and were used for the normalization of the target protein signals. Results are expressed as a percentage of normalized PV/K_v_3.1b/HCN1 levels measured in a mixture of lysates from six WT forebrains that was loaded on all membranes. Representative Western blot signals for PV **(A2)**, K_v_3.1b **(B2)**, and HCN1 **(C2)** are shown. All data are expressed as mean ± SEM.

PV^+^ and VVA^+^ cells were counted independently without crosschecking the other channel to ensure unbiased cell estimates. In the striatum of control mice, ∼90% of PV^+^ cells were also VVA^+^ (**Figure 2A**), whereas in the cortex, the selectivity was slightly lower with ∼75% of PV^+^ cells also being positive for VVA (**Figures 3A and 4A**). Within the pool of VVA^+^ cells, ∼70% and ∼65% of cells in control mice were also identified as PV^+^ in the striatum (**Figure 2A**) and the cortex (**Figures 3A** and **4A**), respectively. Similar values were obtained in previous studies (Ye and Miao, 2013; Filice et al., 2016). The number of PV^+^ cells in the striatum of VPA mice was reduced by ∼15% (*p* = 0.0093) compared to controls (**Figure 2A**). However, there was no difference in the number of VVA^+^ cells between the two groups in the same region (**Figure 2A**), indicating that the number of Pvalb neurons was not decreased in VPA mice. We thus determined the number of double-labeled cells in the striatum. In saline-treated control mice, 71% of VVA^+^ cells were also PV^+^, whereas in VPA mice, this ratio was significantly decreased by ∼15% (*p* = 0.0448) (**Figure 2A**). This hinted that PV protein expression levels might be down-regulated in VPA mice. No difference was seen for PV-positive (PV^+^) cells that also stained positive for VVA in the three investigated regions (PV pool; **Figures 2A, 3A**, and **4A**), suggesting that globally VVA^+^ PNNs were not affected by VPA exposure. Representative immunofluorescence images of the striatum are shown in **Figure 2D**. Although cell estimates obtained by the fractionator method are absolute, we decided to determine the volume of the analyzed ROIs to exclude major macroscopically discernable developmental abnormalities. The volume of the striatum, as measured by the Cavalieri estimator, was not different between VPA and control mice (**Figure 2A**). To certify that PV expression was decreased in VPA mice, PV levels were determined by qRT-PCR and Western blotting. In line with the stereological counts, striatal *Pvalb* mRNA levels were decreased by ∼50% (*p* = 0.0246) in VPA mice compared to control animals (**Figure 2B**). Likewise, PV protein expression levels in the striatum were decreased by ∼30% (*p* = 0.0218) in VPA mice, fully supporting the stereological findings (**Figure 2C**). GAPDH was used to normalize the PV signals on the Western blots (**Figure 2C**). Since all Pvalb neurons in the striatum are GABAergic and moreover express the potassium voltage-gated channel subfamily C member 1 (KCNC1/K_v_3.1) (Chow et al., 1999) and subfamily S member 3 (KCNS3/K_v_9.3) (Georgiev et al., 2012), we quantified transcript levels for glutamic acid decarboxylase 67 (*Gad67*), the major GABA-synthetizing enzyme in the brain, *Kcnc1* and *Kcns3* in VPA and control mice. For all 3 Pvalb neuron markers, transcript levels were not different in the striatum between VPA and control mice (**Figures 5B1,D,F**). Altogether, these results strongly indicated that VPA treatment resulted in decreased striatal PV expression. Reduced PV expression was also shown before in the striatum of PV^+/-^ mice and moreover, in the same region in Shank3B^-/-^ knockout mice (Filice et al., 2016).

### Selected Cortical Regions (mPFC, SSC) of VPA Mice Show Unaltered Pvalb Neuron Numbers

Pvalb neurons in the rat and mouse VPA model have previously been investigated and animals were reported to exhibit a decreased number of PV^+^ cells, considered as the result of Pvalb neuronal loss in the neocortex and the colliculi superior (superficial and intermediate/deep layers), respectively (Gogolla et al., 2009; Dendrinos et al., 2011). To confirm these findings, but at the same time to investigate the alternate possibility that the decrease in PV^+^ neurons was the result of PV down-regulation, we examined two ASD-associated cortical structures, namely SSC and mPFC. Stereological analysis of both regions did not reveal altered numbers of Pvalb neurons in VPA compared to control mice, since neither the number of PV^+^ cells nor the one for VVA^+^ cells was different from control mice (**Figures 3A** and **4A**). Representative immunofluorescence images of the SSC and mPFC are shown in **Figures 3B** and **4B**, respectively. Of note, the volume of the mPFC was slightly increased by ∼20% (*p* = 0.0286) in VPA mice compared to control mice (**Figure 4A**). Indeed, brain overgrowth during infancy is a hallmark of ASD pathophysiology that has been observed in human cases (Courchesne et al., 2011), in VPA mice (Go et al., 2012) and several ASD mouse models including Shank3^-/-^ mice (Ellegood et al., 2015). Nonetheless, calculating the density of Pvalb cells per unit volume did not result in a significantly decreased number of Pvalb neurons in the mPFC of VPA mice. The volume of the SSC was similar between VPA and control mice (**Figure 3A**).

### PV Expression Levels are Unchanged in Forebrain Lysates, but Levels of K_v_3.1b are Decreased, While Levels of HCN1 are Increased

Consistent with IHC stereology results from mPFC and SSC (**Figures 3** and **4**) and global appearance of PV staining in the cortex (data not shown), *Pvalb* mRNA and protein levels from forebrain samples were similar between groups (**Figure 5A1,A2**). To avoid cross-contamination, the forebrain tissue mostly comprising neocortical tissue, but also including the subcortical structures thalamus and pallidus was not further dissected; thus, reduced spatial resolution is a limitation in the qRT-PCR and Western blot analyses. Next, we quantified G*ad67, Kcnc1* (K_v_3.1), and *Kcns3* (K_v_9.3) mRNA levels in the forebrain samples. *Gad67* expression was not decreased in VPA mice; rather slightly increased, supporting the finding that there was no loss of GABAergic interneurons in VPA mice (**Figure 5D**). Moreover, transcript levels of *Kcns3*, which is selectively expressed in Pvalb neocortical neurons, were even slightly, but not significantly (*p* = 0.1095) increased in VPA compared to control mice (**Figure 5F**), in support of an unaltered (certainly not decreased) number of Pvalb neurons. The precise localization of K_v_9.3 giving rise to a *Kcns3* transcript signal in the striatum is currently unknown. Irrespective of its origin, *Kcns3* was unaltered in the VPA mice (**Figure 5F**).

In contrast, forebrain mRNA levels of *Kcnc1* coding for K_v_3.1 were significantly decreased by ∼40% in VPA mice (*p* = 0.0114) (**Figure 5B1**). This decrease was also confirmed at the protein level: K_v_3.1b protein levels determined by Western blot analysis were decreased to a similar extent, i.e., ∼40% reduction (*p* = 0.0002) of K_v_3.1b in forebrain samples of VPA mice compared to controls (**Figure 5B2**), a finding in line with previous results from lysates of SSC of PND15-21 VPA mice (Iijima et al., 2016). We estimate that these results reflect decreased expression of K_v_3.1 channels rather than loss of neurons expressing K_v_3.1, since the overlap between cortical Pvalb neurons (unchanged in mPFC and SSC; **Figures 3A** and **4A**) and K_v_3.1b is >95% (Chow et al., 1999). Since results with respect to Pvalb neurons in VPA mice showed a high similarity with what we had observed before in Shank3B^-/-^ mice, i.e., a significant decrease in striatal PV levels (Filice et al., 2016) and moreover the recent findings that Shank3 mutations resulted in a decrease in hyperpolarization-activated cation (*I*_h_) currents likely being the result of decreased HCN3 and HCN4 levels (Yi et al., 2016), we investigated transcript levels of the various hyperpolarization-activated cyclic nucleotide-gated (HCN) channels in VPA mice including transcript levels of *Hcn1, Hcn2*, and *Hcn4*, the predominant forms expressed in the brain. While *Hcn2* and *Hcn4* transcript levels were unchanged (**Figures 5G,H**); *Hcn1*, which does not co-localize with Pvalb neurons in the neocortex and the basal ganglia (Morris et al., 2004) was significantly up-regulated by ∼40% (*p* = 0.0484) in forebrain samples of VPA mice (**Figure 5C1**). The increase in HCN1 was also verified at the protein level; Western blotting showed a ∼40% increase (*p* = 0.0342) in VPA mice compared to controls (**Figure 5C2**). Interestingly, when we quantitatively determined transcript levels of *Hcn1, Hcn2*, and *Hcn4* in the striatum, no differences were observed between VPA and control mice (**Figures 5C1,G,H**). Finally, we determined mRNA levels of *Kcnc2* (K_v_3.2) to see whether K_v_3.2 quantitatively compensates for the K_v_3.1 deficit. K_v_3.2 is a close relative of K_v_3.1, but co-localizes to a lesser extend with Pvalb neurons (Chow et al., 1999). There was no difference in forebrain mRNA levels of K_v_3.2 between VPA and control mice (**Figure 5E**). This was also true for the striatum (**Figure 5E**). In summary, our results indicate that VPA mice do neither exhibit Pvalb neuron loss nor a decrease in PV expression levels in the forebrain; instead, they display reduced levels of K_v_3.1b in combination with augmented expression of HCN1. The functional implications of these alterations in forebrain regions including the neocortex and the selective down-regulation of PV in the striatum, possibly linked to homeostatic plasticity, are discussed below.

## Discussion

With far more than 100 putative risk genes and additionally epigenetic and environmental risk factors, the pathophysiology of ASD is extremely heterogeneous and complex. One of the major current scientific aims is therefore to find common pathways that link not only different risk genes, but also epigenetic and environmental risk factors. During embryonic development, not only genetic mutations may lead to aberrant brain development, but also external insults may affect the correct migration and differentiation of neuronal precursor cells. This includes VPA shown to impair normal brain development in humans as well as in rodents. Also at the morpho-functional level, VPA mice and rats exhibit ASD-specific abnormalities such as: decreased neuron numbers in different brain areas (Rodier et al., 1996; Lukose et al., 2011; Kataoka et al., 2013), an increase in a postsynaptic form of long-term potentiation (LTP) between pyramidal cells in the somatosensory cortex (Rinaldi et al., 2008), and deficits in inhibitory signaling (Markram et al., 2008; Banerjee et al., 2013). Altered synaptic transmission in VPA mice or rats is of particular interest, since ASD are broadly characterized by synaptic deficits leading to an E/I imbalance in the brain (Rubenstein and Merzenich, 2003). Therefore, it is important to explicitly address the question when, where and how alterations/deficits in neuronal signaling occur in an ASD model. In the VPA model, early postnatal enhancement of NMDA receptor-mediated transmission and increased plasticity in the SSC (Rinaldi et al., 2007) as well as hyper-connectivity and hyper-plasticity of pyramidal synaptic connections in the prefrontal cortex (Rinaldi et al., 2008) have been reported. Intriguingly, in the same study, layer 5 pyramidal neurons in ∼PND14 mice were found to be less excitable in VPA animals compared to controls (Rinaldi et al., 2008), a finding that was confirmed in mice for layer 2/3 pyramidal neurons in the mPFC (Walcott et al., 2011). Since the two developmental deviations (increased NMDA receptor-mediated transmission and hypo-excitability) show the same time line, it was hypothesized that anomalies in one might lead to a homeostatic response to the other (Walcott et al., 2011) according to the concept of homeostatic synaptic plasticity (Turrigiano, 2011). Our observed upregulation of HCN1 therefore might represent a possible mechanism mediating such a transient change in intrinsic neuronal properties leading to hypo-excitability secondary to the observed hyper-connectivity and hyper-plasticity in the mPFC in young mice according to findings reported by Rinaldi (Rinaldi et al., 2008). The difference in intrinsic excitability disappeared in adolescent (>1 month) mice demonstrating the transient nature of plasticity mechanisms (Walcott et al., 2011). In our study, we found increased levels of HCN1 in the cortex of VPA mice. The various HCN channel members HCN1–4 differ in their tissue distribution, within the brain in particular regions and neuron types and in their activation kinetics. They are responsible for generating hyperpolarization-activated *I*_h_ currents, important for controlling membrane resting potentials, input resistance, dendritic integration, synaptic transmission and neuronal excitability (Biel et al., 2009; Benarroch, 2013). Elimination of HCN subunits or blocking of I_h_ currents generally increases dendritic input resistance and leads to hyper-excitability and enhanced neuronal firing (Shah et al., 2004; Huang et al., 2009). Inversely, activity-dependent decrease of excitability through enhancement of *I*_h_ currents is seen in rat hippocampal neurons (Fan et al., 2005). Moreover, suppressed burst-firing and decreased input resistance due to enhanced *I*_h_ currents have been observed in thalamo-cortical neurons in the genetic absence epilepsy rat from Strasbourg (GAERS) model, which shows upregulation of HCN1 protein levels (Kuisle et al., 2006; Cain et al., 2015). Given that HCN1 is prominently expressed in neocortical pyramidal cell dendrites (Moosmang et al., 1999; Lorincz et al., 2002; Morris et al., 2004), increased levels of HCN1 and subsequent enhancement of *I*_h_ currents are likely to lower input resistance and thus might contribute to the hypo-excitability observed in cortical pyramidal neurons of VPA rats during the first month of development. Indeed, membrane input resistance in the mPFC and SSC is slightly, yet not significantly lower in VPA compared to control rats (Rinaldi et al., 2008).

Also altered inhibitory signaling contributes to the pathophysiology of ASD. Impaired pre- and post-synaptic inhibitory transmission in the temporal cortex (Banerjee et al., 2013), decreased inhibition in the lateral amygdala (Markram et al., 2008) and a decrease in the PV^+^ neuron number assumed to be the result of a loss of PV-expressing neurons in the neocortex (Gogolla et al., 2009) and the colliculi superiors (Dendrinos et al., 2011) have been observed in VPA mice and rats. An involvement of the Pvalb neurons in ASD and schizophrenia is nowadays undisputed. However, the question whether Pvalb neurons are indeed lost or simply deficient/reduced in PV protein levels is rarely addressed in an appropriate way, e.g., by using other markers to unequivocally identifying the Pvalb neuron population. Considering that the two possibilities have opposite consequences, this is of great importance: loss of Pvalb neurons leads to decreased inhibition, whereas PV down-regulation has the opposite effect, i.e., by its absence increasing short-term facilitation and thus enhancing inhibition (Schwaller, 2012). Herein we have shown that the number of Pvalb neurons, as determined by the number of VVA^+^ PNN-enwrapped cells, is unchanged in the striatum, mPFC and SSC of VPA mice compared to controls. Thus, the reduced numbers of PV^+^ neurons detected in the striatum of VPA mice reflect decreased PV expression levels, also confirmed at mRNA and protein levels, without any indication for Pvalb neuron loss. While PV protein levels were clearly decreased in the striatum of VPA mice, this didn’t occur in the cortex, where instead altered expression of HCN1 and K_v_3.1b was observed (see below). In neurons, PV is essentially implicated in the subtle modulation of Ca^2+^ signals and moreover involved in regulating the Ca^2+^ homeostasis and subsequently fine-tuning of neuronal signaling (Schwaller, 2012). The absence of PV in PV^-/-^ mice results in enhanced facilitation and modified frequency-specific short-term plasticity at FSI to striatal medium-sized spiny neuron synapses (Orduz et al., 2013). Assuming a similar mechanism in VPA mice, this might represent an adaptive/homeostatic mechanism to strengthen impaired inhibitory signaling in the striatum of VPA mice by enhancing the output of inhibitory Pvalb neurons. Of note, the absence of PV not only affects the output of the FSI, but also entails modifications in synaptic transmission between the cortical afferences and striatal FSI; in PV^-/-^ mice short-term depression of EPSCs is increased in a similar time window (Wohr et al., 2015) as short-term facilitation is increased in PV-FSI (Orduz et al., 2013). We did not detect altered levels of PV in the forebrain samples of VPA mice, in contrast to recent findings by Iijima et al. (2016), who reported a decrease in PV levels by >20% in lysates from neocortical cortex of PND15-21 mice. This apparent discrepancy might be related to the time point of their analyses: in the period from PND15-25, PV protein levels, the number of PV-immunoreactive neurons or of Pvalb neuron structures (e.g., boutons) increase considerably, e.g., seen in mouse or rat cortex (Alcantara et al., 1993; de Lecea et al., 1995; Huang et al., 1999), hippocampus (de Lecea et al., 1995; Chen et al., 2014), and cerebellar Pvalb neurons (Collin et al., 2005). In the reported cases adult levels were reached in the time window of PND24-28, coinciding with the electrophysiological maturation of GABAergic interneurons leveling off by the end of the 4th week of development (Chattopadhyaya et al., 2004; Doischer et al., 2008; Okaty et al., 2009). Yet, the precise trajectory of PV expression levels and of functional Pvalb neuron maturation in mouse SSC and mPFC are currently unknown. For a direct comparison of VPA mice explored in this study with previously investigated models such as Shank1^-/-^, Shank3B^-/-^, as well as PV^+/-^ and PV^-/-^ mice (Filice et al., 2016), the latter having also shown behavioral phenotypes with relevance to all ASD core symptoms at PND25 (Wohr et al., 2015), we selected this time point in our study. Of note, our forebrain lysates of PND25 mice that were used for quantification of RNA and protein levels provide a lower spatial resolution than our results obtained by stereological counts. Interestingly in PND70-80 mice having reached adult PV expression levels with certainty, no differences existed in SSC PV levels between VPA and control mice (Iijima et al., 2016).

Both, mRNA and protein levels of K_v_3.1b were significantly decreased in forebrain extracts of VPA mice as also reported for SSC recently (Iijima et al., 2016). In the mouse cortex, K_v_3.1b channels are exclusively expressed in Pvalb neurons (99% of all PV^+^ neurons are K_v_3.1b^+^ and *vice versa* (Chow et al., 1999)), where they are necessary for maintaining the fast-spiking phenotype of these neurons (Chow et al., 1999; Hernandez-Pineda et al., 1999; Lien and Jonas, 2003). The next closest relative, K_v_3.2, is also expressed in Pvalb neurons, but its distribution is more widespread. Not only is it expressed in other interneuron types, but also in cortical glutamatergic inputs from thalamo-cortical neurons (Chow et al., 1999). K_v_3 channels are voltage-gated K^+^ channels involved in the rapid repolarization of the action potential (AP) mostly in fast-spiking neurons (Lien and Jonas, 2003). K_v_3.1 deficiency, or pharmacologically blocking K_v_3 channels, leads to broadening of AP duration due to a reduced rate of repolarization resulting in a potentiation of transmitter release (Erisir et al., 1999; Porcello et al., 2002; Lien and Jonas, 2003; Jonas et al., 2004; Espinosa et al., 2008). Resulting from the observed decrease of K_v_3.1b in Pvalb neurons leading to after-hyperpolarization (AHP) likely mediated by K_v_3.2 that are characterized by a 2-3 fold slower deactivation kinetics compared to K_v_3.1b, we hypothesize enhanced and likely kinetically slower GABA release from Pvalb neurons. Indeed, mIPSC kinetics in the temporal cortex of VPA rats are significantly slower with respect to both rise and decay times (Banerjee et al., 2013). Likely as a consequence of broadening of AP, fast-frequency firing is severely compromised in neurons from K_v_3.1^-/-^ mice (Erisir et al., 1999; Porcello et al., 2002; Lien and Jonas, 2003). This in turn leads to alterations in oscillatory synchrony patterns, a function normally exerted by Pvalb neurons, resulting in a gamma dys-synchrony phenotype (Joho et al., 1999; Porcello et al., 2002), a feature often observed in ASD (Uhlhaas and Singer, 2007). Of note, reductions in gamma power are observed in hippocampal slices of PV^-/-^ mice *in vitro* (Vreugdenhil et al., 2003), however, the effects of PV-deficiency on gamma oscillations in the cortex *in vivo* are unknown.

Expression of *Pvalb, Hcn1*, and *Kcnc1* genes are subject to activity/experience-dependent regulation mechanisms (Fan et al., 2005; Grabert and Wahle, 2009; Berridge, 2014). Consequently, the observed alterations in expression levels of PV, HCN1, and K_v_3.1 are likely the result of altered brain development and most probably altered neuronal signaling in VPA mice during early postnatal development. The modifications brought about by VPA are brain region-specific and include striatum, mPFC and SSC. However, at the functional level, there appears to exist a certain convergence. Both the decrease in PV in striatal Pvalb neurons, as well as the decrease in K_v_3.1b in forebrain Pvalb neurons might be viewed as homeostatic mechanisms to augment Pvalb neuron-mediated inhibition counteracting the hyper-connectivity and hyper-plasticity observed in mPFC pyramidal neurons (Rinaldi et al., 2008).

Interestingly, the decrease in cortical PV as well as K_v_3.1 expression observed in the SSC of young (PND15–PND21) VPA mice is no longer present in adult (PND70–PND90) mice (Iijima et al., 2016). This would fit to the concept of homeostatic plasticity, which suggests the presence of adaptive/homeostatic regulatory mechanisms in the brain that aim to maintain the stability and functionality of neural circuits when challenged by environmental (e.g., VPA) or other insults during development (Turrigiano, 2011). Such likely mechanisms are operational in HCN1^-/-^ mice; the absence of HCN1 resulted in an increase in background GABA_A_ currents by up-regulating GABA_A_ α5 subunit expression (Chen et al., 2010). According to our study, such homeostatic mechanisms are brain region- and gene-specific in VPA mice. Further studies including functional experiments and moreover determination of expression profiles of, e.g., K_v_3.1b, HCN1 in more precisely defined brain regions (mPFC, SSC) need to be performed to confirm these hypotheses.

Serving as the input structure of the basal ganglia, the striatum receives a great number of sensory inputs and participates in regulating motor control, behavioral flexibility, motivational state, goal-directed learning, and attention. In particular, striatal dysfunction is assumed to underlie repetitive motor behaviors commonly seen in ASD (Fuccillo, 2016). Striatal PV downregulation has previously been described in PV^+/-^ and Shank3B^-/-^ mice (Filice et al., 2016) and both of these models show a robust ASD phenotype including repetitive or stereotyped patterns of behavior (Peca et al., 2011; Wohr et al., 2015). Moreover, alterations in striatal structure or function have been found in human ASD patients (Langen et al., 2007; Estes et al., 2011) and multiple ASD mouse model such as FMR1^-/-^ (Centonze et al., 2008), Shank3^-/-^ (Peca et al., 2011), CNTNAP2^-/-^ (Penagarikano et al., 2011), CNTNAP4^-/-^ (Karayannis et al., 2014), and VPA rats (Schneider et al., 2007). Taken together, striatal PV downregulation represents a promising cellular/morphological phenotype overlapping between different ASD models. It remains to be shown whether alterations in PV, HCN1 and K_v_3.1 are common to other ASD models and/or observed in human ASD subjects and whether they persist into adulthood. If confirmed, the three genes might represent attractive targets for novel therapeutic strategies in ASD.

## Author Contributions

BS conceived the study, participated in the data analyses and in the writing of the manuscript. EL carried out the experiments, performed the statistical analysis and participated in writing of the manuscript. FF participated in setting up stereological experiments and writing of the manuscript. All authors read and approved the final manuscript.

## Conflict of Interest Statement

The authors declare that the research was conducted in the absence of any commercial or financial relationships that could be construed as a potential conflict of interest.
