# Supplementary material for: Prenatal Valproate Exposure Differentially Affects Parvalbumin-Expressing Neurons and Related Circuits in the Cortex and Striatum of Mice
Source: Front Mol Neurosci. 2016 Dec 21;9:150. doi: 10.3389/fnmol.2016.00150 (PMC5174119; doi:10.3389/fnmol.2016.00150)
Supplement: Supplementary file 1 [file Data_Sheet_1.DOCX]

**Supplementary Information**

**
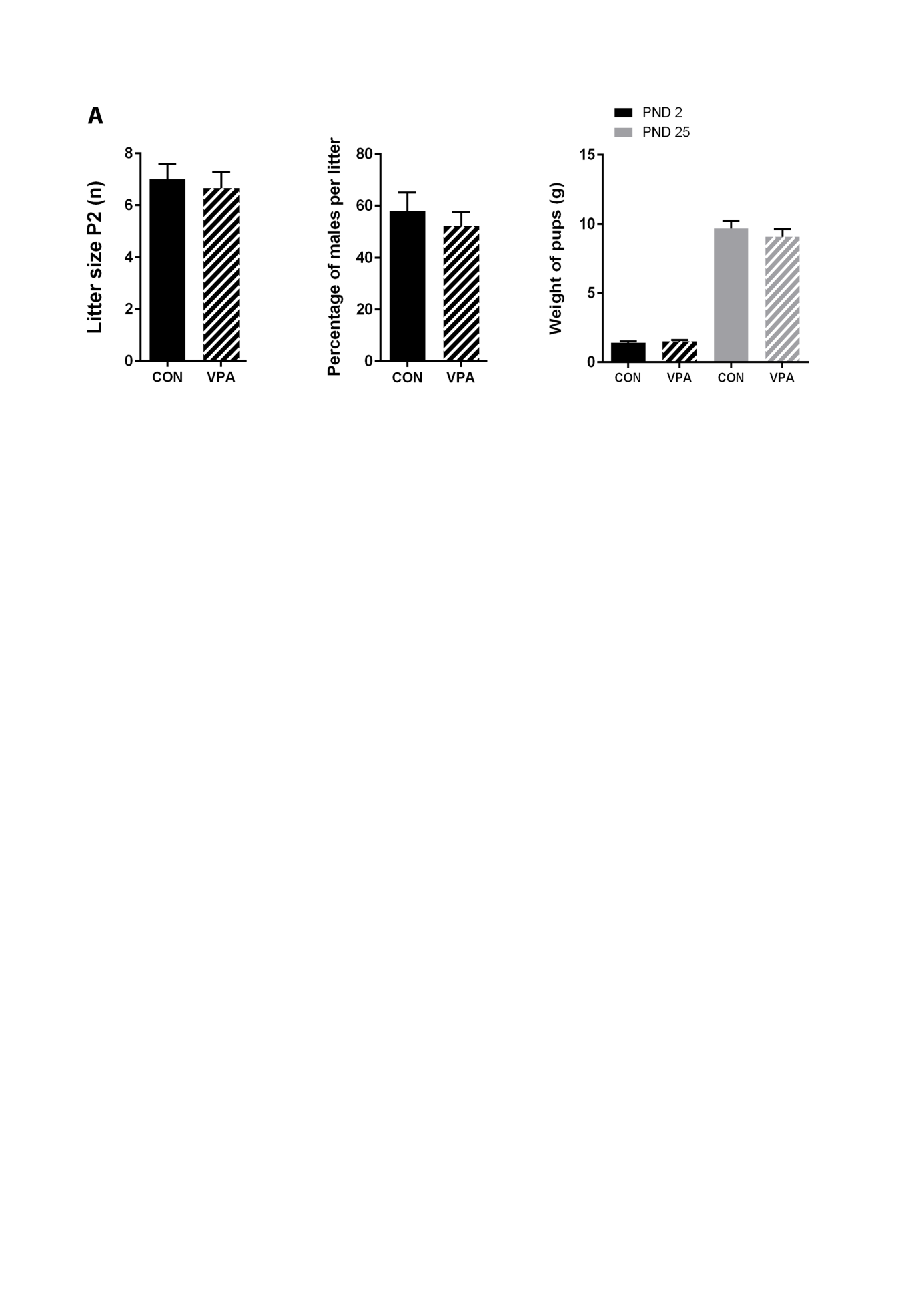
**

**Supplementary Figure 1:** General health parameters of *in utero* saline (CON) and VPA- exposed male mice. **(A)** Left: Mean number of pups per litter. Middle: Sex distribution within litters. Right: Mean weight of litters at PND2 and PND25. None of the parameters was statistically different between control and VPA mice.


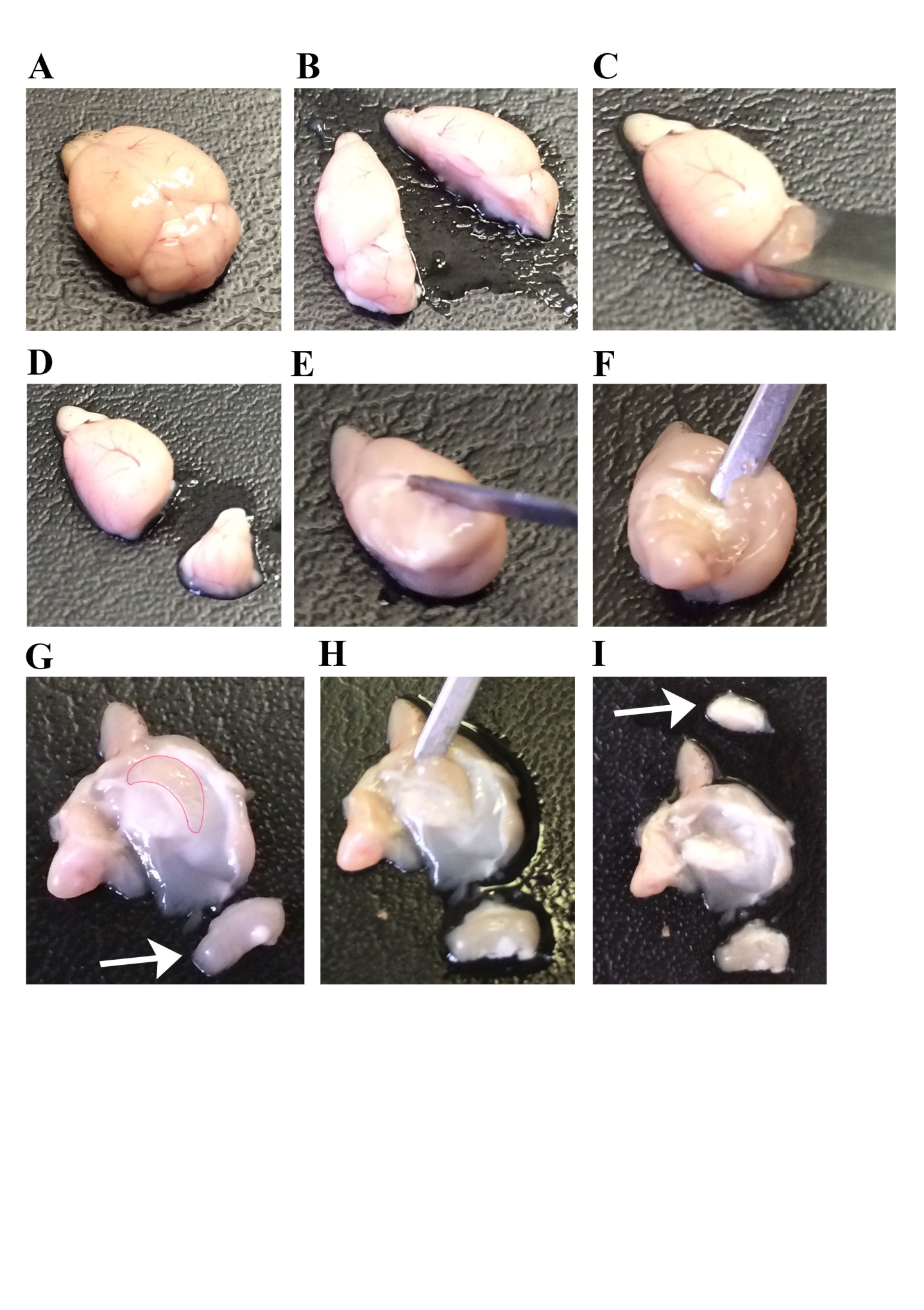


**Supplementary Figure 2:** Dissection of mouse brains for qRT-PCR and Western blot analysis. Immediately after removal, the brains were put in ice-cold 0,9% saline* and cut in half along the midline **(A-B)**. The cerebellum was removed **(C-D)** and the thalamus was separated from the hemisphere along the capsula interna **(E-F)**. In between, the hippocampus was carefully removed (pulled out) from the brain **(F-G).** The arrow in G) points at the hippocampus after removal. Next, the striatum (outlined in bright red in panel G) was carefully removed (pulled out) from the hemisphere using a spatula **(H-I)**. The arrow in panel I) points at the striatum after removal. The remaining parts of the brain (including the thalamus and pallidum) were collected as “forebrain” sample.

*For demonstration purposes, the depicted brain (not included in the study) was not dissected in saline solution.
